# Aqua{5,5′-dimethoxy-2,2-[ethane-1,2-diylbis(nitrilomethylidyne)]diphenolato}nickel(II)

**DOI:** 10.1107/S1600536809039129

**Published:** 2009-10-03

**Authors:** Chunbao Tang

**Affiliations:** a Department of Chemistry, Jiaying University, Meizhou 514015, People’s Republic of China

## Abstract

The title mononuclear nickel(II) complex, [Ni(C_18_H_18_N_2_O_4_)(H_2_O)], possesses crystallographic mirror symmetry. The Ni atom is five-coordinated in a square-pyramidal geometry, with two imine N and two phenolate O atoms of the Schiff base ligand in the square plane, and the water O atom in the axial position. In the crystal, the mol­ecules are linked *via* inter­molecular O—H⋯O hydrogen bonds, forming chains along the *a* axis.

## Related literature

For related structures, see: Angulo *et al.* (2001 ▶); Dey *et al.* (2004 ▶); Edison *et al.* (2004 ▶); Ramadevi *et al.* (2005 ▶); Suh *et al.* (1996 ▶); Tang (2009 ▶).
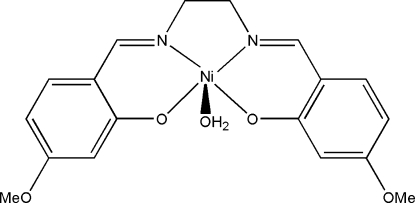


## Experimental

### 

#### Crystal data

[Ni(C_18_H_18_N_2_O_4_)(H_2_O)]
*M* *_r_* = 403.07
Orthorhombic, 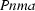

*a* = 8.7698 (3) Å
*b* = 27.0608 (9) Å
*c* = 7.4731 (2) Å
*V* = 1773.5 (1) Å^3^
*Z* = 4
Mo *K*α radiation
μ = 1.13 mm^−1^
*T* = 298 K
0.18 × 0.17 × 0.17 mm

#### Data collection

Bruker SMART CCD area detector diffractometer
Absorption correction: multi-scan (*SADABS*; Sheldrick, 1996 ▶) *T*_min_ = 0.823, *T*_max_ = 0.832
9937 measured reflections
1978 independent reflections
1762 reflections with *I* > 2σ(*I*)
*R*_int_ = 0.023

#### Refinement

*R*[*F* ^2^ > 2σ(*F* ^2^)] = 0.034
*wR*(*F* ^2^) = 0.085
*S* = 1.04
1978 reflections
125 parameters
1 restraint
H atoms treated by a mixture of independent and constrained refinement
Δρ_max_ = 0.39 e Å^−3^
Δρ_min_ = −0.46 e Å^−3^

### 

Data collection: *SMART* (Bruker, 2002 ▶); cell refinement: *SAINT* (Bruker, 2002 ▶); data reduction: *SAINT*; program(s) used to solve structure: *SHELXS97* (Sheldrick, 2008 ▶); program(s) used to refine structure: *SHELXL97* (Sheldrick, 2008 ▶); molecular graphics: *SHELXTL* (Sheldrick, 2008 ▶); software used to prepare material for publication: *SHELXTL*.

## Supplementary Material

Crystal structure: contains datablocks global, I. DOI: 10.1107/S1600536809039129/sj2661sup1.cif

Structure factors: contains datablocks I. DOI: 10.1107/S1600536809039129/sj2661Isup2.hkl

Additional supplementary materials:  crystallographic information; 3D view; checkCIF report

## Figures and Tables

**Table 1:** Hydrogen-bond geometry (Å, °)

*D*—H⋯*A*	*D*—H	H⋯*A*	*D*⋯*A*	*D*—H⋯*A*
O3—H3⋯O1^i^	0.847 (10)	1.969 (17)	2.734 (2)	150 (3)

Symmetry code: (i) 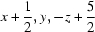.

**Fig. 1.**
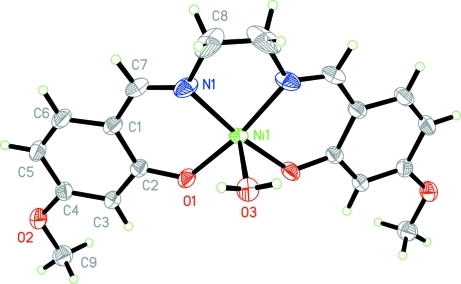
The molecular structure of the title complex. Displacement ellipsoids are drawn at the 30% probability level.

**Fig. 2.**
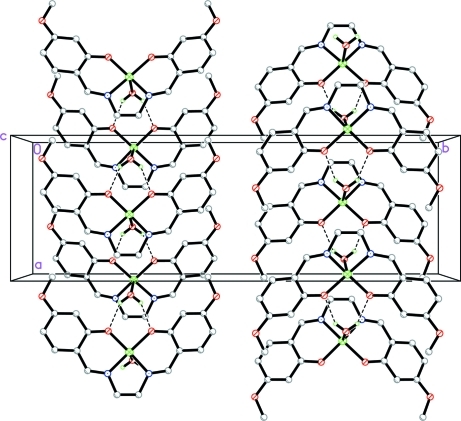
Packing of the title complex, viewed along the c axis. Intermolecular O—H···O hydrogen bonds are shown as dashed lines.

## supplementary crystallographic information

### Comment

Nickel(II) complexes play an important role in both bioinorganic chemistry and coordination chemistry (Suh *et al.*, 1996; Dey *et al.*, 2004; Angulo *et al.*, 2001; Ramadevi *et al.*, 2005; Edison *et al.*, 2004). Recently, the author has reported a nickel(II) complex with a related Schiff base ligand (Tang, 2009). As a continuation of this work, the title mononuclear nickel(II) complex, Fig. 1, is reported here.

The molecule of the title complex possesses crystallographic mirror symmetry. The Ni atom in the complex is five-coordinated by two imine N and two phenolate O atoms of the Schiff base ligand, and by one water O atom, forming a square-pyramidal geometry.

In the crystal structure, the molecules are linked through intermolecular O—H···O hydrogen bonds (Table 1), forming chains along the *a* axis, as shown in Fig. 2.

### Experimental

4-Methoxy-2-hydroxybenzaldehyde (0.2 mmol, 30.5 mg), ethane-1,2-diamine (0.1 mmol, 6.0 mg) and nickel(II) nitrate hexahydrate (0.1 mmol, 29.1 mg) were mixed in a methanol solution (20 ml). The mixture was stirred at room temperature for 30 min to give a green solution. The solution was allowed to stand in air for 5 days, yielding green block-shaped crystals of the title complex.

### Refinement

Water H atoms were located from a difference Fourier map and refined isotropically, with O—H distance restrained to 0.85 (1) Å. Other H atoms were constrained to ideal geometries, with C—H = 0.93–0.97 Å and *U*_iso_(H) = 1.2*U*_eq_(C).

### Crystal data

**Table:**

[Ni(C_18_H_18_N_2_O_4_)(H_2_O)]	*F*(000) = 840
*M**_r_* = 403.07	*D*_x_ = 1.510 Mg m^−^^3^
Orthorhombic, *P**n**m**a*	Mo *K*α radiation, λ = 0.71073 Å
Hall symbol: -P 2ac 2n	Cell parameters from 4326 reflections
*a* = 8.7698 (3) Å	θ = 2.3–29.2°
*b* = 27.0608 (9) Å	µ = 1.13 mm^−^^1^
*c* = 7.4731 (2) Å	*T* = 298 K
*V* = 1773.5 (1) Å^3^	Block, green
*Z* = 4	0.18 × 0.17 × 0.17 mm

### Data collection

**Table:**

Bruker SMART CCD area detector diffractometer	1978 independent reflections
Radiation source: fine-focus sealed tube	1762 reflections with *I* > 2σ(*I*)
graphite	*R*_int_ = 0.023
ω scans	θ_max_ = 27.0°, θ_min_ = 2.8°
Absorption correction: multi-scan (*SADABS*; Sheldrick, 1996)	*h* = −10→11
*T*_min_ = 0.823, *T*_max_ = 0.832	*k* = −34→32
9937 measured reflections	*l* = −9→8

### Refinement

**Table:**

Refinement on *F*^2^	Primary atom site location: structure-invariant direct methods
Least-squares matrix: full	Secondary atom site location: difference Fourier map
*R*[*F*^2^ > 2σ(*F*^2^)] = 0.034	Hydrogen site location: inferred from neighbouring sites
*wR*(*F*^2^) = 0.085	H atoms treated by a mixture of independent and constrained refinement
*S* = 1.04	*w* = 1/[σ^2^(*F*_o_^2^) + (0.0327*P*)^2^ + 1.6368*P*] where *P* = (*F*_o_^2^ + 2*F*_c_^2^)/3
1978 reflections	(Δ/σ)_max_ = 0.001
125 parameters	Δρ_max_ = 0.39 e Å^−^^3^
1 restraint	Δρ_min_ = −0.46 e Å^−^^3^

### Special details

**Table:**

Geometry. All e.s.d.'s (except the e.s.d. in the dihedral angle between two l.s. planes) are estimated using the full covariance matrix. The cell e.s.d.'s are taken into account individually in the estimation of e.s.d.'s in distances, angles and torsion angles; correlations between e.s.d.'s in cell parameters are only used when they are defined by crystal symmetry. An approximate (isotropic) treatment of cell e.s.d.'s is used for estimating e.s.d.'s involving l.s. planes.
Refinement. Refinement of *F*^2^ against ALL reflections. The weighted *R*-factor *wR* and goodness of fit *S* are based on *F*^2^, conventional *R*-factors *R* are based on *F*, with *F* set to zero for negative *F*^2^. The threshold expression of *F*^2^ > σ(*F*^2^) is used only for calculating *R*-factors(gt) *etc*. and is not relevant to the choice of reflections for refinement. *R*-factors based on *F*^2^ are statistically about twice as large as those based on *F*, and *R*- factors based on ALL data will be even larger.

### Fractional atomic coordinates and isotropic or equivalent isotropic displacement parameters (Å^2^)

**Table:**

	*x*	*y*	*z*	*U*_iso_*/*U*_eq_	
Ni1	0.04557 (4)	0.2500	1.04045 (5)	0.03460 (13)	
N1	0.1893 (2)	0.29806 (9)	0.9426 (3)	0.0540 (5)	
O1	−0.10331 (18)	0.30124 (6)	1.0871 (2)	0.0459 (4)	
O2	−0.3611 (2)	0.45495 (6)	1.0277 (2)	0.0558 (5)	
O3	0.1380 (3)	0.2500	1.3276 (3)	0.0467 (5)	
C1	0.0251 (3)	0.36946 (9)	0.9443 (3)	0.0425 (5)	
C2	−0.1014 (3)	0.34714 (8)	1.0324 (3)	0.0379 (5)	
C3	−0.2323 (3)	0.37628 (8)	1.0615 (3)	0.0412 (5)	
H3A	−0.3160	0.3625	1.1197	0.049*	
C4	−0.2387 (3)	0.42474 (9)	1.0054 (3)	0.0440 (5)	
C5	−0.1154 (3)	0.44666 (9)	0.9178 (3)	0.0535 (6)	
H5	−0.1204	0.4793	0.8791	0.064*	
C6	0.0129 (3)	0.41896 (9)	0.8904 (3)	0.0519 (6)	
H6	0.0958	0.4336	0.8336	0.062*	
C7	0.1637 (3)	0.34352 (10)	0.9075 (3)	0.0501 (6)	
H7	0.2418	0.3613	0.8532	0.060*	
C8	0.3344 (3)	0.27498 (13)	0.8944 (7)	0.1204 (18)	
H8A	0.3633	0.2863	0.7760	0.144*	
H8B	0.4119	0.2863	0.9774	0.144*	
C9	−0.4961 (3)	0.43441 (10)	1.1044 (4)	0.0623 (7)	
H9A	−0.5306	0.4073	1.0321	0.093*	
H9B	−0.5740	0.4593	1.1096	0.093*	
H9C	−0.4743	0.4228	1.2231	0.093*	
H3	0.194 (3)	0.2744 (7)	1.355 (4)	0.080*	

### Atomic displacement parameters (Å^2^)

**Table:**

	*U*^11^	*U*^22^	*U*^33^	*U*^12^	*U*^13^	*U*^23^
Ni1	0.02532 (19)	0.0415 (2)	0.0369 (2)	0.000	0.00467 (15)	0.000
N1	0.0328 (10)	0.0682 (14)	0.0611 (13)	−0.0012 (9)	0.0092 (9)	0.0186 (11)
O1	0.0374 (8)	0.0394 (8)	0.0610 (10)	−0.0007 (7)	0.0115 (7)	0.0110 (7)
O2	0.0664 (11)	0.0380 (9)	0.0631 (11)	0.0067 (8)	0.0063 (9)	0.0083 (8)
O3	0.0341 (12)	0.0514 (14)	0.0545 (14)	0.000	−0.0101 (11)	0.000
C1	0.0450 (12)	0.0462 (12)	0.0363 (11)	−0.0125 (10)	0.0011 (9)	0.0016 (10)
C2	0.0390 (11)	0.0389 (11)	0.0360 (10)	−0.0064 (9)	−0.0016 (9)	0.0025 (9)
C3	0.0421 (12)	0.0373 (11)	0.0443 (12)	−0.0050 (9)	0.0034 (10)	0.0022 (9)
C4	0.0555 (14)	0.0387 (12)	0.0378 (11)	−0.0029 (10)	−0.0022 (10)	0.0006 (9)
C5	0.0708 (17)	0.0381 (12)	0.0518 (14)	−0.0100 (12)	0.0048 (13)	0.0077 (11)
C6	0.0578 (15)	0.0502 (14)	0.0478 (13)	−0.0181 (12)	0.0072 (12)	0.0067 (11)
C7	0.0397 (12)	0.0629 (16)	0.0478 (13)	−0.0120 (11)	0.0051 (10)	0.0133 (12)
C8	0.0448 (16)	0.104 (3)	0.212 (5)	0.0225 (16)	0.056 (2)	0.079 (3)
C9	0.0617 (16)	0.0539 (15)	0.0713 (18)	0.0134 (13)	0.0116 (15)	0.0120 (14)

### Geometric parameters (Å, °)

**Table:**

Ni1—O1^i^	1.9363 (16)	C2—C3	1.410 (3)
Ni1—O1	1.9363 (16)	C3—C4	1.378 (3)
Ni1—N1^i^	1.953 (2)	C3—H3A	0.9300
Ni1—N1	1.953 (2)	C4—C5	1.396 (3)
Ni1—O3	2.294 (2)	C5—C6	1.367 (4)
N1—C7	1.278 (3)	C5—H5	0.9300
N1—C8	1.463 (3)	C6—H6	0.9300
O1—C2	1.308 (3)	C7—H7	0.9300
O2—C4	1.359 (3)	C8—C8^i^	1.352 (7)
O2—C9	1.428 (3)	C8—H8A	0.9700
O3—H3	0.847 (10)	C8—H8B	0.9700
C1—C6	1.403 (3)	C9—H9A	0.9600
C1—C2	1.424 (3)	C9—H9B	0.9600
C1—C7	1.430 (3)	C9—H9C	0.9600
			
O1^i^—Ni1—O1	91.47 (9)	O2—C4—C3	124.6 (2)
O1^i^—Ni1—N1^i^	91.48 (8)	O2—C4—C5	114.4 (2)
O1—Ni1—N1^i^	168.38 (9)	C3—C4—C5	121.0 (2)
O1^i^—Ni1—N1	168.38 (9)	C6—C5—C4	118.3 (2)
O1—Ni1—N1	91.48 (8)	C6—C5—H5	120.8
N1^i^—Ni1—N1	83.49 (13)	C4—C5—H5	120.8
O1^i^—Ni1—O3	94.00 (7)	C5—C6—C1	122.9 (2)
O1—Ni1—O3	94.00 (7)	C5—C6—H6	118.6
N1^i^—Ni1—O3	97.00 (8)	C1—C6—H6	118.6
N1—Ni1—O3	97.00 (8)	N1—C7—C1	125.6 (2)
C7—N1—C8	120.9 (2)	N1—C7—H7	117.2
C7—N1—Ni1	127.17 (17)	C1—C7—H7	117.2
C8—N1—Ni1	111.69 (19)	C8^i^—C8—N1	115.27 (16)
C2—O1—Ni1	127.95 (14)	C8^i^—C8—H8A	108.5
C4—O2—C9	118.01 (19)	N1—C8—H8A	108.5
Ni1—O3—H3	115 (2)	C8^i^—C8—H8B	108.5
C6—C1—C2	118.6 (2)	N1—C8—H8B	108.5
C6—C1—C7	118.6 (2)	H8A—C8—H8B	107.5
C2—C1—C7	122.8 (2)	O2—C9—H9A	109.5
O1—C2—C3	118.17 (19)	O2—C9—H9B	109.5
O1—C2—C1	123.9 (2)	H9A—C9—H9B	109.5
C3—C2—C1	117.9 (2)	O2—C9—H9C	109.5
C4—C3—C2	121.2 (2)	H9A—C9—H9C	109.5
C4—C3—H3A	119.4	H9B—C9—H9C	109.5
C2—C3—H3A	119.4		

Symmetry codes: (i) *x*, −*y*+1/2, *z*.

### Hydrogen-bond geometry (Å, °)

**Table:**

*D*—H···*A*	*D*—H	H···*A*	*D*···*A*	*D*—H···*A*
O3—H3···O1^ii^	0.85 (1)	1.97 (2)	2.734 (2)	150 (3)

Symmetry codes: (ii) *x*+1/2, *y*, −*z*+5/2.
